# The impact of the Covid-19 pandemic on mental and physical health in Denmark – a longitudinal population-based study before and during the first wave

**DOI:** 10.1186/s12889-021-11472-7

**Published:** 2021-07-18

**Authors:** Marie Weinreich Petersen, Thomas Meinertz Dantoft, Jens Søndergaard Jensen, Heidi Frølund Pedersen, Lisbeth Frostholm, Michael Eriksen Benros, Tina Birgitte Wisbech Carstensen, Eva Ørnbøl, Per Fink

**Affiliations:** 1 The Research Clinic for Functional Disorders and Psychosomatics, Aarhus University hospital, Universitetsbyen 21-23, 8000 Aarhus C, Denmark; 2 Center for Clinical Research and Prevention, Bispebjerg and Frederiksberg Hospital, The Capital Region of Denmark, Copenhagen, Denmark; 3 Department of Clinical Medicine, University of Aarhus, Aarhus, Denmark; 4 Mental Health Centre Copenhagen, The Capital Region of Denmark, Copenhagen, Denmark; 5 Department of Immunology and Microbiology, Faculty of Health and Medical Sciences, University of Copenhagen, Blegdamsvej 3B, 2200 Copenhagen, Denmark

**Keywords:** Covid-19, Corona virus, Mental health, Physical health, Emotional distress, Somatic symptoms, Illness worry, Health anxiety, Longitudinal cohort study

## Abstract

**Background:**

In the continuation of the first wave of the Covid-19 outbreak in Denmark, unprecedented restrictions with great impact on the citizen’s everyday life were implemented. The objectives of this study were to investigate the influence of the Covid-19 pandemic on mental and physical health in the Danish population during the spring 2020 first wave outbreak and lockdown.

**Methods:**

A sample from the adult Danish population (*n* = 2190) were included. Self-reported measures of illness worry (Whiteley-6-R), emotional distress (SCL-90), and physical symptom load (SLC-90) were obtained before and during the first wave of the pandemic and compared with Wilcoxon signed-rank tests. Impact of covariates on physical and mental health was evaluated with ordinal regression analyses. Results from a tailored questionnaire regarding the Covid-19 pandemic were presented to explore the direct impact of the pandemic.

**Results:**

We only found minor increases in illness worry, emotional distress and physical symptom load (*0–1 points difference, p ≤ 0.007*) during the Covid-19 pandemic compared to before the pandemic. Sex, age, education, and physical disease were not associated with illness worry, emotional distress, or physical symptom load. Overall, the participants were trustful in the authorities’ recommendations and felt that they managed the pandemic and the restrictions to a great extent despite that some expected great/major future consequences of the pandemic.

**Conclusions:**

This study suggested that the first wave of the Covid-19 pandemic only had minor impact on mental and physical health in the Danish general population. Future studies should address the impact of the second wave of the pandemic and the renewed implementation of the concomitant restrictions.

**Supplementary Information:**

The online version contains supplementary material available at 10.1186/s12889-021-11472-7.

## Introduction

Covid-19 was first observed in December 2019 and quickly evolved into a worldwide pandemic during 2020. Nations worldwide reacted by imposing restrictions and lockdowns in order to stop the SARS-CoV-2 virus from spreading to fast. The first Danish Covid-19 positive case was reported on February 272,020, and on March 7 the Danish government confirmed 27 cases of Covid-19 who had been infected within Danish borders [1]. On March 11, WHO declared the Covid-19 to be a worldwide pandemic, which led the Danish government to launch a set of initiatives in collaboration with the Danish health authorities to prevent the Covid-19 virus to dissipate in the Danish society and to shield the resources in the Danish health care system [2]. This constituted several unprecedented restrictions with great impact on the Danish citizens’ everyday life: Lockdown of educational institutions and childcare facilities, public employees were instructed to work from home, private companies were encouraged to instruct their employees to work from home as well, gatherings of more than 100 (and later 10) individuals were prohibited, lockdown of non-essential health care, a total lockdown of restaurants, health clubs, sport activities etc., and closing of the Danish borders [3, 4]. Denmark was the first European country to impose a temporary boarder closure; however, according to the Oxford Covid-19 Government Response Tracker, the stringency level imposed by the Danish authorities during the spring 2020 was rather similar to the global government response [5].

Evidence on a great impact on physical and psychological well-being as well as social behavior has been established during previous severe epidemics with SARS-CoV.1 and H1N1 [6–9]. Initial studies into the Covid-19 pandemic have also shown negative impact of the outbreak and the following restrictions on physical and mental health in general populations, resulting in e.g. increased levels of stress, anxiety, and symptoms of depression, [10–16] as well as negative economic consequences and societal disruptions [17]. However, a review and meta-analysis concluded that the psychological impact from the Covid-19 lockdown on general populations was small and highly heterogeneous [18]. In Denmark, more attempts to estimate the impact of the Covid-19 pandemic have been carried out [19–21] (Andersen PB, Christensen HR, Jacobsen BA, Kühle L, Cour Pl, Pedersen HF, et al. Covid-19 – Religion and existential wellbeing 2020. Accepted for publication Religionsvidenskabeligt Tidsskrift 2020; Unpublished). Danish studies into the Covid-19 pandemic have shown varying results when it comes to the pandemic’s influence on physical and mental health in the Danish population [22–24]. Importantly, Danish studies concerning the first wave of the pandemic have either been done in a cross-sectional design or in longitudinal designs but with no possibility of including paired data obtained before the outbreak of Covid-19.

In the current population-based longitudinal study, we included paired data on physical and mental health obtained before and during the first wave of the Covid-19 pandemic in Denmark. The objectives were to investigate the Covid-19 pandemic’s influence on everyday life and physical and mental health in the Danish population.

## Methods

### Study sample

**Fig. 1:**
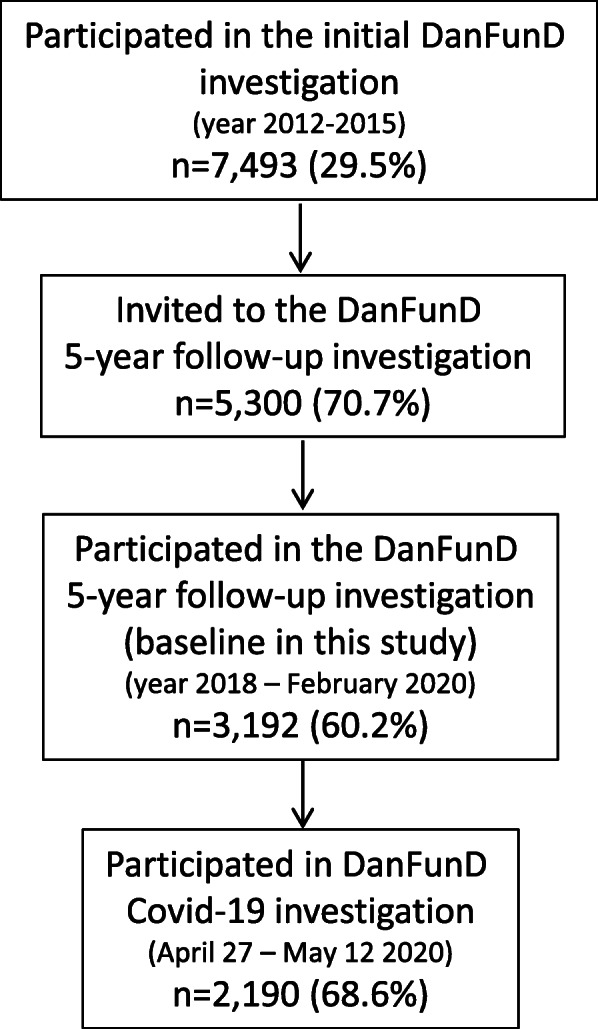
Flow of study participants. DanFunD=Danish Study of Functional Disorders

The study included data from the Danish Study of Functional Disorders (DanFunD) five-year follow-up cohort and included data obtained at two different time points (Fig. 1). Initial data collection for DanFunD took place between 2012 and 2015 [25]. Here, a total of 25,369 men and women aged 18–72 years, born in Denmark and living in the western part of greater Copenhagen, were randomly obtained from the nationwide Danish registries and invited to participate. A total of 7493 (29.5%) agreed to participate.

Data collection for the DanFunD five-year follow-up investigation (the baseline investigation in the current study) was initiated in 2018 and was planned to be finished in the end of 2020. All the initial 7493 participants were invited to participate in the same order they had participated in the initial DanFunD investigation. The inclusion was, however, paused from March 122,020 due to the COVID-19 pandemic. At this point, 3192 (60.2%) participants had been re-examined.

All 3192 re-examined participants from the DanFunD five-year follow-up cohort were asked to complete an additional questionnaire survey during the first wave of the COVID-19 pandemic between April 27 and May 12. Surveys were sent out by the secure digital mailbox ‘e-boks’ and postal reminding letters were sent out after seven days.

### Primary measures

Three primary outcome measures were included. The measures were obtained both before and during the first wave of the Covid-19 pandemic:

1. Illness worry: Illness worry was measured with the six-item Whiteley-6-R [26]. Responses are recorded on a 5-point rating scale ranging from “not at all” to “a great deal”. The index generates a sum score ranging from 0 to 24 with increasing scores indicating higher levels of illness worry.

2. Emotional distress: Emotional distress was measured with eight items from the 90-item Symptom Check List (SCL-90) addressing impairment of overall worries, depression, and anxiety [27–29]. Responses were recorded on a 5-point rating scale ranging from “not at all” to “a great deal”. The sum score ranging from 0 to 32, and higher scores indicated elevated levels of emotional distress.

3. Physical symptom load: Physical symptom load was measured with the 12-item somatization subscale (SCL-SOM) of the SCL-90. Responses were recorded on a 5-point rating scale ranging from “not at all” to “a great deal”. The sum score ranged from 0 to 48, and higher scores indicated elevated levels of physical symptom load.

For all primary measures, the time frame covered was 12 months for the first investigation conducted before the Covid-19 pandemic and six weeks for the follow-up investigation conducted during the first wave of the Covid-19 pandemic.

### Secondary measures

Data on the first investigation conducted before the Covid-19 pandemic and the follow-up investigation conducted during the first wave of the Covid-19 pandemic both included, among others, validated questions on social factors, social network, and severe physical disease (i.e. presence of at least one self-reported diagnosis of cancer, diabetes, myocardial infarction, other heart disease, stroke, obstructive pulmonary disease) [30, 31].

All questionnaires addressed the past 12 months for the five-year follow-up investigation (baseline) and six weeks for the follow-up investigation during the first wave of the Covid-19 pandemic.

For the follow-up investigation conducted during the first wave of the Covid-19 pandemic, a range of additional tailored questions were applied to explore the direct impact of the pandemic on the participants (i.e. family life, work, social and economic worries, worries on being infected or to infect others, medical examination, whether they themselves or family members had been infected, and trust in the government and health care system). Questions from the Covid-19 tailored questionnaire are displayed in Appendix A.

### Statistical analyses

#### Primary measures - descriptives

Analyses on sample characteristics and comparison of mental and physical measures before and during the Covid-19 pandemic were performed in STATA version 16.0 [32]. Descriptive statistics were presented as mean and standard deviations (SD) or as medians and interquartile ranges (IQR) depending on the distribution of the continuous variables. For categorical variables, frequencies with percentages were shown. Responders of the Covid-19 follow-up investigation were compared to non-responders on age, sex, cohabitation, and education with Pearson’s Chi-squared tests.

Mental and physical measures before and during the Covid-19 pandemic were presented as median values and IQR and compared with Wilcoxon signed-rank tests.

#### Primary measures – regression analyses and model check

A total of 12 ordinal regression analyses were conducted in R Studio version 1.2.5033 (using the “rms” package) [33]. Linear regression modelling was not chosen for the analyses as the model assumptions were not fulfilled. Checking the models, the QQ-plots showed non-linearity, and plots of the residuals against the fitted values showed heterogeneity of variance. The ordinal regression analyses were performed with three primary outcomes (illness worry, emotional distress, and physical symptom load) and four primary independent variables (sex (female/male), age (continuous), education (no/ 0 years, short/< 3 years, medium /3–4 years, long/> 4 years), and chronic disease (yes/no)).

Age was modelled using restricted cubic splines with five knots at the 5th, 27.5th, 50th, 72.5th, and 95th percentiles according to the recommendations by Harrell [34] to avoid the strong assumption of a linearity.

In all models, the adjusted median score and the corresponding 95% confidence intervals (CI) of the primary outcome of interest were estimated at different values of the primary independent variable [35]. The ordinal regression models all used the logit function as link function, and the fit of the models was examined graphically using residual plots as proposed in Harrell [34].

Associations between the primary outcomes and each of the primary independent variables were tested with ANOVA Wald Chi-Squared Tests.

Correction of multiple testing was performed with Bonferroni correction with the critical significance level set at 0.05 and 12 tests. Therefore, a significance level ≤ 0.004 indicated rejection of the null hypothesis of no difference.

#### Choice of confounders in the analyses of primary measures

Potential confounders included in the analyses were identified using directed acyclic graphs (DAGs) constructed in the browser-based programme DAGitty version 3.0 [36]. The choice of confounders was based on the theory by Pearl et al. [37]. First, a range of variables, obtainable in our data, were chosen based in exiting literature. A variable was only included in the DAGs as a confounder if it influenced both the primary outcome and the primary independent variable. Therefore, different confounders were evaluated on and included in each of the 12 regression analyses and they varied across primary outcomes: Analyses on primary outcomes and sex were adjusted for baseline values of the primary outcome, age, worry about the pandemic, and presence of physical disease. Analyses on primary outcomes and age were adjusted for baseline values of the primary outcome, worry about the pandemic, presence of physical disease, and trust in the government. Analyses on primary outcomes and education were adjusted for baseline values of the primary outcome. Analyses on primary outcomes and presence of physical disease were adjusted for baseline values of the primary outcome, age, and trust in the government.

#### Secondary measures

Secondary measures on self-perceived consequences of the Covid-19 pandemic were presented as descriptive statistics. Depending on data distribution, continuous variables were presented as means with SD or medians with IQR. Categorical variables were presented as proportions.

## Results

### Sample characteristics

A total of 2190 (68.6%) participated in the study with complete data before and during the first wave of the pandemic. At baseline, median age was 63 years (IQR: 54–69); 53.4% were women. Most participants (82.2%) were cohabiting and had at least 3 years of further education (77.4%). A total of 6.7% reported to have a poor health, 26.9% reported to have received at least one diagnosis of severe physical disease (cancer, diabetes, stroke, myocardial infarction, other heart disease, obstructive pulmonary disease) at one point in life, and 10.9% reported to have received at least one diagnosis of either depression or anxiety. More details of sample characteristics are displayed in Appendix B.

Regarding sex distribution and presence of physical and mental conditions, responders for the follow-up Covid-19 investigation did not differ from non-responders. However, compared to responders, non-responders were younger (median age: 58, IQR: 50–68, *p < 0.0001*), fewer were cohabiting (78.8%), *p = 0.02*), and fewer had shorter further education (*p < 0.0001*).

### Impact of the Covid-19 pandemic on mental and physical health

**Fig. 2:**
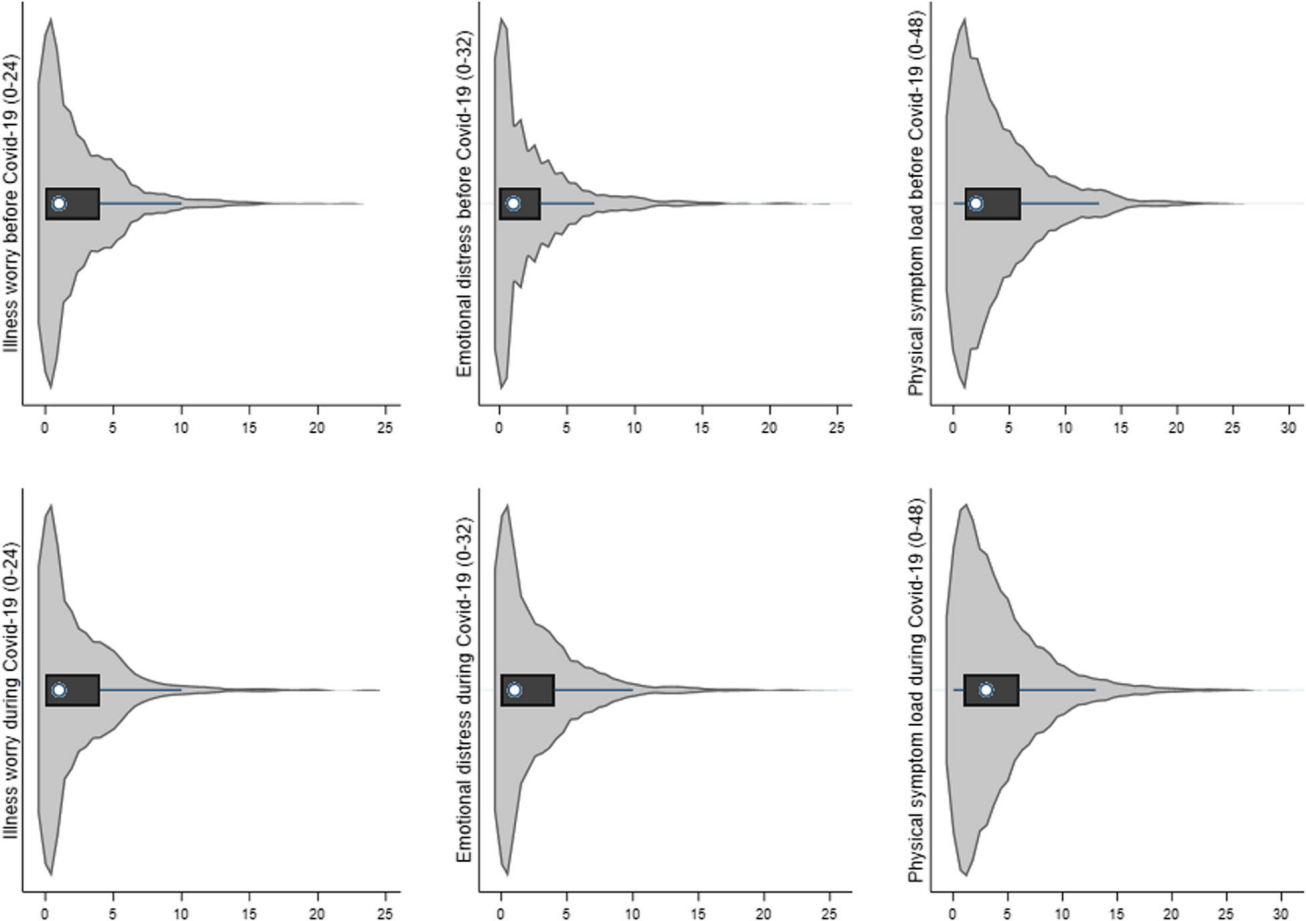
Mental and physical health before and during the Covid-19 pandemic. Level of illness worry, emotional distress, and physical symptom load before and during the Covid-19 pandemic in Denmark

We only found minor worsening of illness worry (median score: 1, IQR: 0–4 vs. median score: 1, IQR: 0–4, *Z = -2.69, p = 0.007)*, emotional distress (median score: 1, IQR: 0–4 vs. median score: 1, IQR: 0–3, *Z = 6.45, p < 0.0001)*, and physical symptom load (median score: 3, IQR: 1–6 vs. median score: 2, IQR: 1–6, *Z = 3.90, p = 0.0001)* during the Covid-19 pandemic as compared to before the pandemic (Fig. 2).

### Association between sex, age, education, and physical disease and mental and physical health during the Covid-19 pandemic

Generally, despite some indication of association between sex, age, education, and physical disease and illness worry, emotional distress, and physical symptom load from the statistical tests (*p-values ranged from < 0.0001 to 0.82*) (Table 1), only minor associations with these covariates was found.

**Table 1:** Association between sex, age, education, and physical disease and health outcomes (*n* = 2190)

	Illness worry	Emotional distress	Physical symptom load
	ANOVA Wald Statistics (Chi-square (df), *p*)		
Sex	*X*^*2*^ [1]=6.96, *p = 0.008*	*X*^*2*^ [1]=23.02, ***p < 0.0001***	*X*^*2*^ [1]=1.85, *p = 0.17*
Age	*X*^*2*^ [4]*=*1.56, *p = 0.82*	*X*^*2*^ [4]=7.97, *p = 0.09*	*X*^*2*^ [4]=17.76, ***p = 0.001***
Education	*X*^*2*^ [3]=5.93, *p = 0.11*	*X*^*2*^ [3]=5.74, *p = 0.12*	*X*^*2*^ [3]=9.78, *p = 0.02*
Physical disease	*X*^*2*^ [1]=4.59, *p = 0.03*	*X*^*2*^ [1]=3.9, *p = 0.05*	*X*^*2*^ [1]=28.79, ***p < 0.0001***

Bold letters indicate rejection of the null hypothesis of no difference after adjusting for multiple testing.

**Fig. 3:**
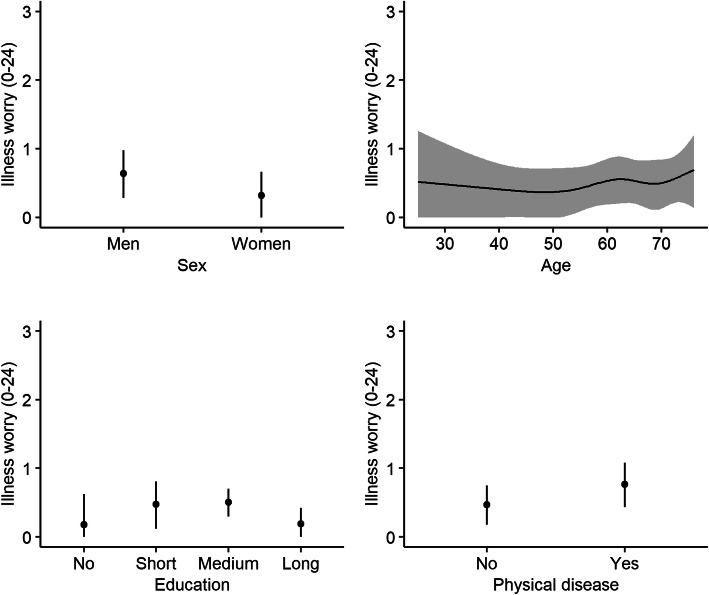
Illness worry during the Covid-19 pandemic. Associations (reported as adjusted median values with 95% confidence intervals) between illness worry and sex, age, education, and physical disease during the Covid-19 pandemic. Illness worry was measured with the Whiteley-6-R [26]. Education: long= > 4 years, medium = 3–4 years, short = < 3 years, no = 0 years

Illness worry was slightly increased in men (median: 0.48, 95% CI: 0.21–0.74 vs. 0.23, 95% CI: 0.0–0.48, Chi^2^ = 6.96, *p = 0.08*) and those having physical disease (median: 0.57, 95% CI: 0.32–0.12 vs. 0.34, 95% CI: 0.12–0.55, Chi^2^ = 4.59, *p = 0.03*) (Fig. 3).

**Fig. 4:**
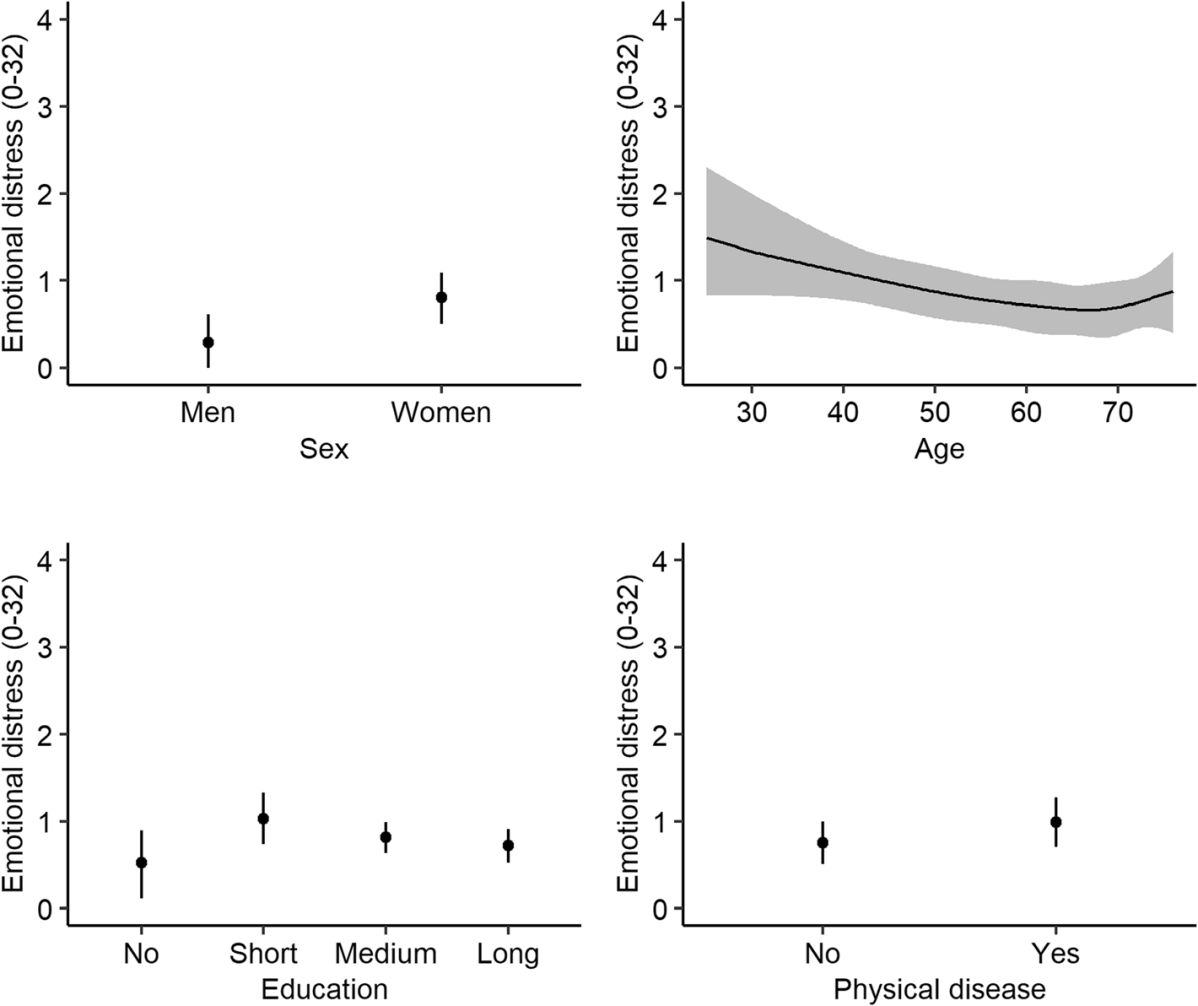
Emotional distress during the Covid-19 pandemic. Associations (reported as adjusted median values with 95% confidence intervals) between emotional distress and sex, age, education, and physical disease during the Covid-19 pandemic. Emotional distress was measured with eight items from the 90-item Symptom Check List addressing impairment of overall worries, depression, and anxiety [27, 28]. Education: long= > 4 years, medium = 3–4 years, short = < 3 years, no = 0 years

Emotional distress was slightly increased in women (median: 0.59, 95% CI: 0.37–0.82 vs. 0.19 95% CI: 0–0.4, Chi^2^ = 23.02, *p < 0.0001*) and those having physical disease (median: 0.74 95% CI: 0.52–0.98 vs. 0.55, 95% CI: 0.36–0.74, Chi^2^ = 3.9, *p = 0.05*) (Fig. 4).

**Fig. 5:**
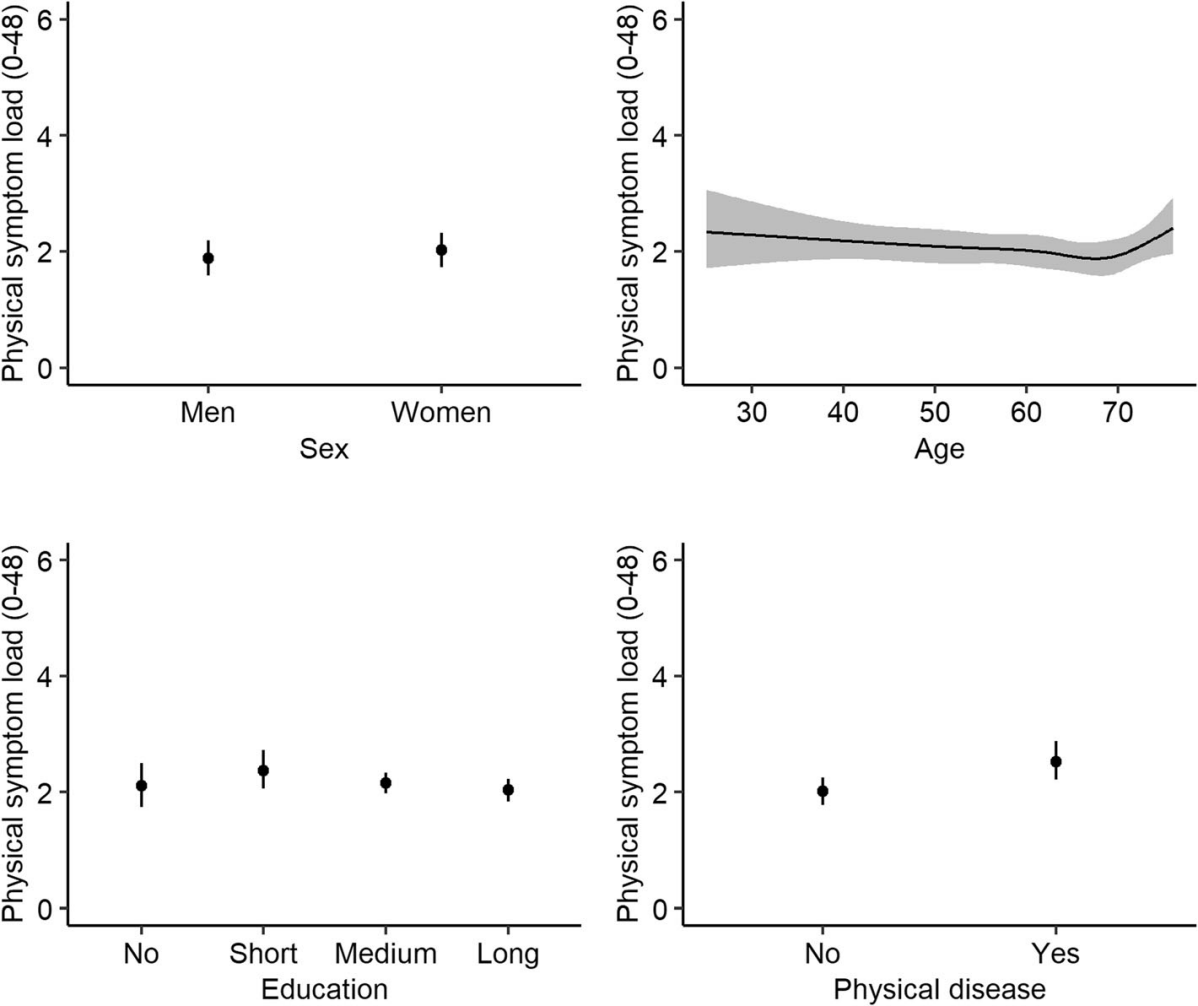
Physical symptom load during the Covid-19 pandemic. Associations (reported as adjusted median values with 95% confidence intervals) between physical symptom load and sex, age, education, and physical disease during the Covid-19 pandemic. Physical symptom load was measured with the somatization subscale from the 90-item Symptom Check List [27]. Education: long= > 4 years, medium = 3–4 years, short = < 3 years, no = 0 years

A minor association between education and physical symptom load was found with the highest physical symptom load in participants with short/< 3 years of education (median: 2.49, 95% CI: 2.07–2.9, Chi^2^ = 9.78, *p = 0.02*) (Fig. 5). As expected, participants with physical disease had higher physical symptom load compared to participants without physical disease (median: 2.7, 95% CI: 2.3–3.1 vs. 1.8, 95% CI: 1.5–2.1, Chi^2^ = 28.79, *p < 0.0001*) (Fig. 5).

### Self-perceived consequences of the Covid-19 pandemic

Covid-19-related descriptive characteristics are displayed in Table 2. Generally, participants had some concerns about the pandemic and some worries about being infected or infecting others. Less than 17% had experienced Covid-19 related symptoms, and a minority had been tested for Covid-19. Only five participants reported to have an active Covid-19 infection at study time and they all experienced mild symptoms. Only one participant had been hospitalized with Covid-19: A severe case with 28 days of hospitalization on life support. Almost 20% had been placed in quarantine, either by own choice or by the authorities. While 14% were acquainted with some that had been infected, only a minority knew someone who had been hospitalized because of Covid-19 or who had passed away with Covid-19.

**Table 2:** Covid-19-related health (*n* = 2190)

Participants’ own illness related to Covid-19	
General concerns about Covid-19 (1–10); mean (SD)	5.0 (2.4)
Have experienced symptoms of Covid-19; % (n)	16.7 (365)
Have been in contact with the health care system because of Covid-19 symptoms; % (n)	4.4 (96)
Have been tested for Covid-19; negative % (n)/ positive % (n)	6.1 (134) / 0.4 (9)
Suspect to have been infected [1–10]; median (IQR)	1 (1–2)
Worried about being infected [1–10]; mean (SD)	4.5 (2.7)
Worried about getting seriously ill if infected (0–10); median (IQR)	3 (2–6)
Worried about infecting others [1–10]; median (IQR)	5 (2–7)
Been placed in quarantine – by authorities; % (n)	2.3 (51)
Been placed in quarantine – by own choice; % (n)	17.8 (390)
**Covid-19-related illness among family and friends**	
Worried about others getting infected [1–10]; median (IQR)	6 (3–8)
Personal acquaintances have been infected; % (n)	14.2 (311)
Personal acquaintances have been hospitalized; % (n)	3.5 (77)
Personal acquaintances have died; % (n)	1.3 (29)
**Government and health authorities’ recommendations**	
Obey the recommendations from health authorities [1–10]; median (IQR)	10 (9–10)
Trust the recommendations from health authorities [1–10]; median (IQR)	9 (8–10)
Agree with the restrictions given by the government [1–10]; median (IQR)	9 (8–10)
**The consequences of the pandemic**	
Get emotionally affected by the pandemic [1–10]; median (IQR)	3 (2–5)
Believe the pandemic to have significant consequences in future life [1–10]; median (IQR)	5 (3–7)
Participants’ own management of restrictions in work life [1–10]; median (IQR)	9 (8–10)
Participants’ own management of restrictions in social life [1–10]; median (IQR)	9 (8–10)
Feel more lonely during the Covid-19 pandemic [1–10]; median (IQR)	3 (1–6)
**Participants’ everyday life during the Covid-19 pandemic**	
Feel challenged in everyday life [1–10]; median (IQR)	5 (4–5)
Feel challenged because of children being at home [1–10]; median (IQR)	1 (1–5)

*SD* standard deviation, *IQR* interquartile range

The full Covid-19 tailored questionnaire is displayed in Appendix A

Participants followed the Covid-19-related restrictions to a great extent, and they were very trustful in the recommendations given by the authorities. Asking the participants if they felt that the recommendations and restrictions from the health authorities were exaggerated, 984 (44.9%) felt that they were not exaggerated at all (scoring 1 on a 1–10 scale). A total of 840 participants (38.4%) had complete trust (scored 10 on a 1–10 scale) in the capacity of the health care system to manage the situation.

Participants were generally not that affected emotionally by the Covid-19 pandemic, and they felt that they managed the restrictions in social and work life to a great extent. For a few participants, the Covid-19 pandemic had had more significant negative impact on their work life: 2% had lost their job, 1.4% had been sent home without receiving salary, and 4.4% had suffered economic consequences in their private companies. Generally, to some extent the participants believed that the pandemic would have significant consequences in the future.

## Discussion

To our knowledge, this is the first study to investigate the impact of the Covid-19 pandemic on the Danish general population in a longitudinal study design where paired data obtained before and during the first wave of the Covid-19 pandemic in the spring 2020 was included and compared. We only found minor worsening of illness worry, emotional distress, and physical symptom load during the Covid-19 pandemic as compared to before the pandemic. Sex, age, education, and presence of physical disease did not seem to influence physical or mental health during the pandemic. Generally, participants had some concerns about the pandemic, they followed the restrictions given by the government, and they had trust in the health authorities in managing the situation.

The results from the current study are in line with some other studies indicating stable levels regarding psychological well-being [23], worries and quality of life [24], and stress, anxiety, and depression [38] during the first wave outbreak and lockdown. These studies were, however, either performed in cross-sectional designs [24] or compared data obtained at other time points than the present study: One compared data at two time points in the initial phase of the outbreak in China (late January-late February) [38] and one at two time points during the spring 2020 (March–April) in Denmark [23].

Other general population-based studies have indicated higher levels of depression [11, 12, 14, 22, 39, 40], emotional distress [13, 41, 42], anxiety [11, 14, 22, 39, 40], and somatic symptom load [11] during the Covid-19 pandemic. Furthermore, associations between poor mental health and female sex [11–14, 22, 39, 41], young age [11–14, 41], low education [11, 12], and presence of physical symptoms [39] have been shown.

Discrepancies between the present study and the above mentioned studies may be caused by methodological differences as some of the studies were conducted in cross-sectional designs [11, 14, 39, 41] which makes it difficult to establish the obtained high levels of mental and physical distress as actual consequences of the pandemic. Some other studies used longitudinal designs but did not compare the same individuals with paired analyses as in the present study [12, 13, 22]. Furthermore, cultural and social differences may also be the reason for the discrepancies: Compared to some other countries, Denmark is a socioeconomically advantaged country and provides a social safety net which secures each Danish individual with economic stability and equal access to welfare benefits and health care regardless of social position. During the first wave of the pandemic, government-induced help packages were made available in Denmark, and hence, most people may not yet have felt significant economic consequences. Therefore, the Danish general population may not have been that affected by the pandemic at study time as other countries with less social security and economic safety net. Furthermore, the level of trust and confidence in the government and authorities among the Danish population was high during the first wave of the Covid-19 pandemic [43], which may also buffer potential stress reactions in the population [44].

As to mental and physical health change over time and in the current study, only data from the first wave of the pandemic in the spring 2020 were included. By that time, Denmark had the pandemic under control, the number of infected and hospitalized individuals was low, and the time perspective of the pandemic was unknown. Previous research has shown that prolonged isolation and quarantine are associated with public mental health problems and psychiatric manifestations [9, 17, 45, 46]. We may therefore have seen more negative impact from the pandemic later on during the second wave, where tiredness started to occur and the restrictions to some extent were questioned by the public. Also, the present study included a sample of well-educated individuals with a high median age of 63 years, and this group has in previous studies shown to be less affected by the pandemic than younger age groups [11–14, 39].

### Strengths and limitations

One of the strengths of the current study is the large number of participants sampled from the general adult population, comprising almost equal proportions of both sexes and with a life span of 54 years. Another major strength is the longitudinal study design which allows us to compare mental and physical health measures before and during the first wave of the Covid-19 pandemic. Last, the inclusion of well-known measures of both mental and physical health constitutes a strength.

Some limitations also need to be addressed. First, the participants in the current study had a high median age and high educational level, and they differed from non-responders on these parameters. This possible selection bias may have caused underestimation on the influence of the pandemic on mental and physical health measures. Second, the included data from before the first wave of the Covid-19 pandemic were gathered throughout a two-year period, hence, it does not comprise an actual measure at one point in time from just before the breakout, but it does, however, represent the health status of the general population in a time of no pandemic. Third, from the included data, we cannot completely rule out that other aspects than the Covid-19 pandemic may influence on the physical and mental health measures.

## Conclusion

The current study suggests that the first wave of the Covid-19 pandemic in the spring 2020 only had minor impact on mental and physical health in the Danish general population. However, future studies should address the impact of the renewed outbreak of the second wave of the pandemic and the implementation of the concomitant restrictions.

## Supplementary Information

**Additional file 1.**

## Data Availability

The datasets used and analysed during the current study are available from the corresponding author on reasonable request.

## Abbreviations

DanFunD: Danish Study of Functional Disorders
SCL-90: 90-item Symptom Check List
SCL-SOM: the SCL-90 somatization subscale
SD: Standard Deviation
IQR: Inter Quartile Range
CI: Confidence Interval
DAGs: Directed Acyclic Graphs
