# Supplementary material for: The impact of the Covid-19 pandemic on mental and physical health in Denmark – a longitudinal population-based study before and during the first wave
Source: BMC Public Health. 2021 Jul 18;21:1418. doi: 10.1186/s12889-021-11472-7 (PMC8286431; doi:10.1186/s12889-021-11472-7)
Supplement: Supplementary file 1 — Additional file 1. [file 12889_2021_11472_MOESM1_ESM.docx]

**Appendix A: Covid-19 questionnaire**

**______________________________________________________**

| **I. Own Corona-related illness** |
| --- |

1. Overall, how much do you worry about the Corona epidemic?

*Please tick the most appropriate number:*

| 1 | 2 | 3 | 4 | 5 | 6 | 7 | 8 | 9 | 10 |
| --- | --- | --- | --- | --- | --- | --- | --- | --- | --- |
| 🞏  Not at all | 🞏 | 🞏 | 🞏 | 🞏 | 🞏 | 🞏 | 🞏 | 🞏 | 🞏  A great deal |

1. Since the Corona virus outbreak in Denmark, have you had any symptoms of Covid-19 infection or symptoms that you think could stem from it?

| 🞏 | Yes | |
| --- | --- | --- |
| 🞏 | No |  |

1. Have you been in contact with your doctor or called the emergency number (1813) because of Covid-19-related symptoms?

| 🞏 | Yes | |
| --- | --- | --- |
| 🞏 | No |  |

1. Have you been tested for Corona virus?

| 🞏 | Yes, the test came out negative | |
| --- | --- | --- |
| 🞏 | Yes, the test came out positive |  |
| 🞏 | Yes, but the test came out inconclusive |  |
| 🞏 | No |  |

1. If possible, would you have preferred to be tested?

| 🞏 | Yes | |
| --- | --- | --- |
| 🞏 | No |  |

1. Do you currently have a Covid-19 infection?

| 🞏 | Yes, with mild symptoms | |
| --- | --- | --- |
| 🞏 | Yes, with moderate symptoms |  |
| 🞏 | Yes, with severe symptoms |  |
| 🞏 | No |  |

1. When you were infected with Covid-19, did you then have

| 🞏 | Mild symptoms? | |
| --- | --- | --- |
| 🞏 | Moderate symptoms? |  |
| 🞏 | Severe symptoms? |  |

1. Do you believe that you are in high risk of serious illness with hospitalization if you get an Covid-19 infection?

*Please tick the most appropriate number:*

| 1 | 2 | 3 | 4 | 5 | 6 | 7 | 8 | 9 | 10 |
| --- | --- | --- | --- | --- | --- | --- | --- | --- | --- |
| 🞏  Not at all | 🞏 | 🞏 | 🞏 | 🞏 | 🞏 | 🞏 | 🞏 | 🞏 | 🞏  To a great extent |

1. Have you been hospitalized because of Covid-19 symptoms?

| 🞏 | Yes | |
| --- | --- | --- |
| 🞏 | No |  |

1. How many days?

|  |  |
| --- | --- |

1. Have you been admitted to intensive care?

| 🞏 | Yes | |
| --- | --- | --- |
| 🞏 | No |  |

1. Were you on life support?

| 🞏 | Yes | |
| --- | --- | --- |
| 🞏 | No |  |

1. Before you got infected, were you then worried about being infected?

*Please tick the most appropriate number:*

| 1 | 2 | 3 | 4 | 5 | 6 | 7 | 8 | 9 | 10 |
| --- | --- | --- | --- | --- | --- | --- | --- | --- | --- |
| 🞏  Not at all | 🞏 | 🞏 | 🞏 | 🞏 | 🞏 | 🞏 | 🞏 | 🞏 | 🞏  To a great extent |

1. Do you suspect being or having been infected with Covid-19 (without having been tested?)

*Please tick the most appropriate number:*

| 1 | 2 | 3 | 4 | 5 | 6 | 7 | 8 | 9 | 10 |
| --- | --- | --- | --- | --- | --- | --- | --- | --- | --- |
| 🞏  Not at all | 🞏 | 🞏 | 🞏 | 🞏 | 🞏 | 🞏 | 🞏 | 🞏 | To a great extent |

1. Are you worried about getting infected with Corona virus?

*Please tick the most appropriate number:*

| 1 | 2 | 3 | 4 | 5 | 6 | 7 | 8 | 9 | 10 |
| --- | --- | --- | --- | --- | --- | --- | --- | --- | --- |
| 🞏  Not at all | 🞏 | 🞏 | 🞏 | 🞏 | 🞏 | 🞏 | 🞏 | 🞏 | 🞏  To a great extent |

1. Do you believe that you are in high risk of serious illness with hospitalization if you get an Covid-19 infection?

*Please tick the most appropriate number:*

| 1 | 2 | 3 | 4 | 5 | 6 | 7 | 8 | 9 | 10 |
| --- | --- | --- | --- | --- | --- | --- | --- | --- | --- |
| 🞏  Not at all | 🞏 | 🞏 | 🞏 | 🞏 | 🞏 | 🞏 | 🞏 | 🞏 | 🞏  To a great extent |

1. Have you been worried about getting severely ill if you got infected?

*Please tick the most appropriate number:*

| 1 | 2 | 3 | 4 | 5 | 6 | 7 | 8 | 9 | 10 |
| --- | --- | --- | --- | --- | --- | --- | --- | --- | --- |
| 🞏  Not at all | 🞏 | 🞏 | 🞏 | 🞏 | 🞏 | 🞏 | 🞏 | 🞏 | 🞏  To a great extent |

1. Are you worried about the risk of infecting others – even if you do not feel ill?

*Please tick the most appropriate number:*

| 1 | 2 | 3 | 4 | 5 | 6 | 7 | 8 | 9 | 10 |
| --- | --- | --- | --- | --- | --- | --- | --- | --- | --- |
| 🞏  Not at all | 🞏 | 🞏 | 🞏 | 🞏 | 🞏 | 🞏 | 🞏 | 🞏 | 🞏  To a great extent |

1. Are you currently in or have you been ordered quarantine by the health authorities because of Covid-19?

| 🞏 | Yes | |
| --- | --- | --- |
| 🞏 | No |  |

1. Are you currently in or have you been in self-quarantine because of Covid-19?

| 🞏 | Yes | |
| --- | --- | --- |
| 🞏 | No |  |

1. Since 1 March 2020, have you been hospitalized for other reasons than Covid-19 virus?

| 🞏 | Yes | |
| --- | --- | --- |
| 🞏 | No |  |

1. For what reason were you hospitalized?

Please state the reason:

____________________

1. Since 1 March 2020, have you been acutely ill for other reason than Covid-19 virus?

🞏 No

🞏 Yes, infectious disease

🞏 Yes, other disease

🞏 Don't know

| 1. **Illness among family and friends** |
| --- |

1. Are you currently or have you been worried about someone you know getting infected with Corona virus?

*Please tick the most appropriate number:*

| 1 | 2 | 3 | 4 | 5 | 6 | 7 | 8 | 9 | 10 |
| --- | --- | --- | --- | --- | --- | --- | --- | --- | --- |
| 🞏  Not at all | 🞏 | 🞏 | 🞏 | 🞏 | 🞏 | 🞏 | 🞏 | 🞏 | 🞏  To a great extent |

1. Has someone in your social circle been tested positive for Corona virus?

🞏 Yes

🞏 No

1. (More than one option can be chosen)

🞏 Close relative (e.g. mother, father, siblings, grandparents)

🞏 Other relatives (e.g. uncle, aunt)

🞏 Friend

🞏 Colleague

🞏 Neighbour

🞏 Others

🞏 No

1. Has someone in your social circle been hospitalized because of Corona virus? (more than one option can be chosen)

🞏 Close relative (e.g. mother, father, siblings, grandparents)

🞏 Other relatives (e.g. uncle, aunt)

🞏 Friend

🞏 Colleague

🞏 Neighbour

🞏 Others

🞏 No

1. Has someone in your social circle passed away because of Corona virus? (more than one option can be chosen)

🞏 Close relative (e.g. mother, father, siblings, grandparents)

🞏 Other relatives (e.g. uncle, aunt)

🞏 Friend

🞏 Colleague

🞏 Neighbour

🞏 Others

🞏 No

| 1. **Recommendations from the authorities** |
| --- |

1. Do you comply with the latest recommendations from the health authorities (e.g. social distancing, avoiding handshakes and crowds)?

*Please tick the most appropriate number:*

| 1 | 2 | 3 | 4 | 5 | 6 | 7 | 8 | 9 | 10 |
| --- | --- | --- | --- | --- | --- | --- | --- | --- | --- |
| 🞏  Not at all | 🞏 | 🞏 | 🞏 | 🞏 | 🞏 | 🞏 | 🞏 | 🞏 | 🞏  To a great extent |

1. Do you have faith in the health authorities' recommendations?

*Please tick the most appropriate number:*

| 1 | 2 | 3 | 4 | 5 | 6 | 7 | 8 | 9 | 10 |
| --- | --- | --- | --- | --- | --- | --- | --- | --- | --- |
| 🞏  Not at all | 🞏 | 🞏 | 🞏 | 🞏 | 🞏 | 🞏 | 🞏 | 🞏 | 🞏  To a great extent |

1. Do you trust that the healthcare system is able to manage the situation?

*Please tick the most appropriate number:*

| 1 | 2 | 3 | 4 | 5 | 6 | 7 | 8 | 9 | 10 |
| --- | --- | --- | --- | --- | --- | --- | --- | --- | --- |
| 🞏  Not at all | 🞏 | 🞏 | 🞏 | 🞏 | 🞏 | 🞏 | 🞏 | 🞏 | 🞏  To a great extent |

1. For a period of time, the government has partially closed down society (e.g. sending home public employees, closing schools and day care centres, recommending working from home). Do you concur with these initiatives as part of the strategy against the Corona virus?

*Please tick the most appropriate number:*

| 1 | 2 | 3 | 4 | 5 | 6 | 7 | 8 | 9 | 10 |
| --- | --- | --- | --- | --- | --- | --- | --- | --- | --- |
| 🞏  Not at all | 🞏 | 🞏 | 🞏 | 🞏 | 🞏 | 🞏 | 🞏 | 🞏 | 🞏  To a great extent |

1. Do you believe that the recommendations from the health authorities and the government are exaggerated?

*Please tick the most appropriate number:*

| 1 | 2 | 3 | 4 | 5 | 6 | 7 | 8 | 9 | 10 |
| --- | --- | --- | --- | --- | --- | --- | --- | --- | --- |
| 🞏  Not at all | 🞏 | 🞏 | 🞏 | 🞏 | 🞏 | 🞏 | 🞏 | 🞏 | 🞏  To a great extent |

| 1. **Consequences of the epidemic** |
| --- |

1. Are you emotionally affected by the Corona epidemic (e.g. does it make you feel angry, scared, worried, or depressed)?

*Please tick the most appropriate number:*

| 1 | 2 | 3 | 4 | 5 | 6 | 7 | 8 | 9 | 10 |
| --- | --- | --- | --- | --- | --- | --- | --- | --- | --- |
| 🞏  Not affected at all | 🞏 | 🞏 | 🞏 | 🞏 | 🞏 | 🞏 | 🞏 | 🞏 | 🞏  Greatly affected |

1. For how long do you think the Corona epidemic will last?

*Please tick the most appropriate number:*

| 1 | 2 | 3 | 4 | 5 | 6 | 7 | 8 | 9 | 10 |
| --- | --- | --- | --- | --- | --- | --- | --- | --- | --- |
| 🞏  Short time (weeks) | 🞏 | 🞏 | 🞏 | 🞏 | 🞏 | 🞏 | 🞏 | 🞏 | 🞏  Very long time (one year or more) |

1. Do you think the Corona epidemic will have consequences in your future life?

*Please tick the most appropriate number:*

| 1 | 2 | 3 | 4 | 5 | 6 | 7 | 8 | 9 | 10 |
| --- | --- | --- | --- | --- | --- | --- | --- | --- | --- |
| 🞏  No conse-quences at all | 🞏 | 🞏 | 🞏 | 🞏 | 🞏 | 🞏 | 🞏 | 🞏 | 🞏  Very severe consequences |

1. If you are studying or working, to what extent do you think you manage the Corona-related restrictions in your study/work life?

*Please tick the most appropriate number:*

| 1 | 2 | 3 | 4 | 5 | 6 | 7 | 8 | 9 | 10 |
| --- | --- | --- | --- | --- | --- | --- | --- | --- | --- |
| 🞏  Not at all | 🞏 | 🞏 | 🞏 | 🞏 | 🞏 | 🞏 | 🞏 | 🞏 | 🞏  To a great extent |

🞏 I am neither studying or working

1. To what extent do you think you manage the Corona-related restrictions in your social life?

*Please tick the most appropriate number:*

| 1 | 2 | 3 | 4 | 5 | 6 | 7 | 8 | 9 | 10 |
| --- | --- | --- | --- | --- | --- | --- | --- | --- | --- |
| 🞏  Not at all | 🞏 | 🞏 | 🞏 | 🞏 | 🞏 | 🞏 | 🞏 | 🞏 | 🞏  To a great extent |

1. Do you fear that the Corona epidemic will return, e.g. in the autumn?

*Please tick the most appropriate number:*

| 1 | 2 | 3 | 4 | 5 | 6 | 7 | 8 | 9 | 10 |
| --- | --- | --- | --- | --- | --- | --- | --- | --- | --- |
| 🞏  Not at all | 🞏 | 🞏 | 🞏 | 🞏 | 🞏 | 🞏 | 🞏 | 🞏 | 🞏  To a great extent |

1. Do you fear for your own private economy because of the Corona epidemic?

*Please tick the most appropriate number:*

| 1 | 2 | 3 | 4 | 5 | 6 | 7 | 8 | 9 | 10 |
| --- | --- | --- | --- | --- | --- | --- | --- | --- | --- |
| 🞏 | 🞏 | 🞏 | 🞏 | 🞏 | 🞏 | 🞏 | 🞏 | 🞏 | 🞏 |
| No Small Moderate Severe consequences consequences consequences consequences | | | | | | | | | |

1. Do you fear for the national or global economy because of the Corona epidemic?

*Please tick the most appropriate number:*

| 1 | 2 | 3 | 4 | 5 | 6 | 7 | 8 | 9 | 10 |
| --- | --- | --- | --- | --- | --- | --- | --- | --- | --- |
| 🞏 | 🞏 | 🞏 | 🞏 | 🞏 | 🞏 | 🞏 | 🞏 | 🞏 | 🞏 |
| No Small Moderate Severe consequences consequences consequences consequences | | | | | | | | | |

1. Have you watched the news about the Corona virus epidemic?

*Please tick the most appropriate number:*

| 1 | 2 | 3 | 4 | 5 | 6 | 7 | 8 | 9 | 10 |
| --- | --- | --- | --- | --- | --- | --- | --- | --- | --- |
| 🞏 | 🞏 | 🞏 | 🞏 | 🞏 | 🞏 | 🞏 | 🞏 | 🞏 | 🞏 |
| Not at all To a great extent | | | | | | | | | |

1. When the Corona epidemic is over, do you think it will change the way we interact as individuals, either positively or negatively?

*Please tick the most appropriate number:*

| 1 | 2 | 3 | 4 | 5 | 6 | 7 | 8 | 9 | 10 |
| --- | --- | --- | --- | --- | --- | --- | --- | --- | --- |
| 🞏 | 🞏 | 🞏 | 🞏 | 🞏 | 🞏 | 🞏 | 🞏 | 🞏 | 🞏 |
| Mostly  positively No change Mostly   negatively | | | | | | | | | |

1. Do you feel more lonely during the Corona epidemic?

*Please tick the most appropriate number:*

| 1 | 2 | 3 | 4 | 5 | 6 | 7 | 8 | 9 | 10 |
| --- | --- | --- | --- | --- | --- | --- | --- | --- | --- |
| 🞏 | 🞏 | 🞏 | 🞏 | 🞏 | 🞏 | 🞏 | 🞏 | 🞏 | 🞏 |
| Not at all To a great   extent | | | | | | | | | |

| 1. **Everyday life during the epidemic** |
| --- |

1. What work- or study-related consequences have the Corona epidemic and the subsequent restrictions had for you (more options can be chosen)?

□ I work/study much more

□ I have lost my job

□ I have been sent home and still receiving salary

□ I have been sent home without receiving salary

□ I work/study from home

□ I have suffered economic consequences in my private company

□ No consequences (also if you are not attached to the labour market, e.g. senior citizens)

□ Other

1. Do you live with someone?

□ Yes, alone with child/children

□ Yes, with spouse/cohabitant partner and child/children

□ Yes, with spouse/cohabitant partner and no child/children

□ Yes, in shared accommodation

□ No

□ Yes, other

1. What work- or study related consequences have the Corona epidemic and the subsequent restrictions had for your spouse/cohabitant partner (more options can be chosen)?

□ He/she works/studies much more

□ He/she has lost his/her job

□ He/she has been sent home, still receiving salary

□ He/she have been sent home without receiving salary

□ He/she works/studies from home

□ He/she has suffered economic consequences in his/her private company

□ No consequences (also if he/she is not attached to the labour market, e.g. senior citizens)

□ Other

1. How do you experience your life together during the Corona epidemic?

*Please tick the most appropriate number:*

| 1 | 2 | 3 | 4 | 5 | 6 | 7 | 8 | 9 | 10 |
| --- | --- | --- | --- | --- | --- | --- | --- | --- | --- |
| 🞏 | 🞏 | 🞏 | 🞏 | 🞏 | 🞏 | 🞏 | 🞏 | 🞏 | 🞏 |
| It is nice, we have Unchanged We are very challenged  become closer | | | | | | | | | |

1. Have you felt distressed having your child/children at home?

*Please tick the most appropriate number:*

| 1 | 2 | 3 | 4 | 5 | 6 | 7 | 8 | 9 | 10 |
| --- | --- | --- | --- | --- | --- | --- | --- | --- | --- |
| 🞏 | 🞏 | 🞏 | 🞏 | 🞏 | 🞏 | 🞏 | 🞏 | 🞏 | 🞏 |
| Not at all To a great extent | | | | | | | | | |

**Appendix B: Sample characteristics**

| **Sample characteristics (n=2,190)** | |
| --- | --- |
|  | |
| **Sex**; n (%) |  |
| Women | 1,169 (53.4) |
| Men | 1,021 (46.6) |
|  |  |
| **Age**; n (%) |  |
| 23-39 | 97 (4.4) |
| 40-49 | 221 (10.1) |
| 50-59 | 569 (26.0) |
| 60-77 | 1303 (59.5) |
|  |  |
| **Cohabitation**; n (%) |  |
| Yes | 1,797 (82.2) |
| No | 389 (17.8) |
|  |  |
| **Marital status**; n (%) |  |
| Married | 1,594 (72.8) |
| Not married | 308 (14.1) |
| Divorced/separated | 205 (9.4) |
| Widower | 77 (3.5) |
|  |  |
| **Vocational training**; n (%) |  |
| 0 years | 173 (7.9) |
| 1-2 years | 277 (12.7) |
| 3-4 years | 944 (43.1) |
| > 4 years | 751 (34.3) |
|  |  |
| **Employment**; n (%) |  |
| Currently employed | 1,501 (68.5) |
| Previously employed | 654 (29.9) |
| Never been employed | 21 (1.0) |
|  |  |
| **Self-perceived health**; n (%) |  |
| Excellent | 245 (11.2) |
| Very good | 982 (44.8) |
| Good | 817 (37.3) |
| Fair | 141 (6.4) |
| Poor | 5 (0.2) |
|  |  |
| **Severe physical disease**; n (%) |  |
| Cancer | 166 (7.6) |
| Diabetes | 87 (4.0) |
| Stroke | 54 (2.5) |
| Myocardial infarction | 40 (1.8) |
| Other heart disease | 112 (5.1) |
| Obstructive pulmonary disease | 24 (1.1) |
|  |  |
| **Mental disorders**; n (%) |  |
| Depression | 206 (9.4) |
| Anxiety | 64 (2.9) |
|  |  |
